# Point-of-care wound visioning technology: Reproducibility and accuracy of a wound measurement app

**DOI:** 10.1371/journal.pone.0183139

**Published:** 2017-08-17

**Authors:** Sheila C. Wang, John A. E. Anderson, Robyn Evans, Kevin Woo, Benjamin Beland, Denis Sasseville, Linda Moreau

**Affiliations:** 1 Department of Medicine, Division of Dermatology, McGill University, Montreal, Quebec, Canada; 2 Department of Psychology, York University, Toronto, Ontario, Canada; 3 Wound Care Centre, Women’s College Hospital, Toronto, Ontario, Canada; 4 School of Nursing, Queen’s University, Kingston, Ontario, Canada; 5 Faculty of Medicine, McGill University, Montreal, Quebec, Canada; IDI, Istituto Dermopatico dell'Immacolata, ITALY

## Abstract

**Background:**

Current wound assessment practices are lacking on several measures. For example, the most common method for measuring wound size is using a ruler, which has been demonstrated to be crude and inaccurate. An increase in periwound temperature is a classic sign of infection but skin temperature is not always measured during wound assessments. To address this, we have developed a smartphone application that enables non-contact wound surface area and temperature measurements. Here we evaluate the inter-rater reliability and accuracy of this novel point-of-care wound assessment tool.

**Methods and findings:**

The wounds of 87 patients were measured using the Swift Wound app and a ruler. The skin surface temperature of 37 patients was also measured using an infrared FLIR^™^ camera integrated with the Swift Wound app and using the clinically accepted reference thermometer Exergen DermaTemp 1001. Accuracy measurements were determined by assessing differences in surface area measurements of 15 plastic wounds between a digital planimeter of known accuracy and the Swift Wound app. To evaluate the impact of training on the reproducibility of the Swift Wound app measurements, three novice raters with no wound care training, measured the length, width and area of 12 plastic model wounds using the app. High inter-rater reliabilities (ICC = 0.97–1.00) and high accuracies were obtained using the Swift Wound app across raters of different levels of training in wound care. The ruler method also yielded reliable wound measurements (ICC = 0.92–0.97), albeit lower than that of the Swift Wound app. Furthermore, there was no statistical difference between the temperature differences measured using the infrared camera and the clinically tested reference thermometer.

**Conclusions:**

The Swift Wound app provides highly reliable and accurate wound measurements. The FLIR^™^ infrared camera integrated into the Swift Wound app provides skin temperature readings equivalent to the clinically tested reference thermometer. Thus, the Swift Wound app has the advantage of being a non-contact, easy-to-use wound measurement tool that allows clinicians to image, measure, and track wound size and temperature from one visit to the next. In addition, this tool may also be used by patients and their caregivers for home monitoring.

## Introduction

Wound measurement is important not only as a means for monitoring wound progression but also for predicting wound healing. Clinical studies have shown that when healing normally, a wound’s area should be 20–40% smaller after 2–4 weeks of treatment[1]. Problematic wounds which take longer to heal need to be identified at the earliest possible stage because delays increase the difficulty of treatment and healing[2, 3]. The most common method for measuring wounds is the ruler-based method, which measures longest length head-to-toe and longest perpendicular width side-to-side[4]. This method has been shown to overestimate wound area by at least 44%[5], especially for wounds with irregular shapes or those that curve around the body [5, 6]. Measuring wounds using digital photography is more accurate than the ruler method [6, 7]. However, calculating wound area by tracing the wound edge from an uploaded digital photo on a computer is more time-consuming and costly than the ruler method; the costs of setting up and maintaining digital photographic management systems range from $2000-$4000 USD, not including the personnel to take the digital photographs, analyse the images and organize the files [8]. Therefore, this process is often impractical in clinical settings. More recently, wound measurement devices with custom software have been developed for computerized photogrammetry and three-dimensional imaging. These include hand-held devices and larger stereo photographic systems equipped with multiple cameras and projection units with up-front costs ranging from $800 to $5000 USD and the need for a trained operator [9].

Recent technological advances in smartphone applications have opened up new solutions to the infrastructure and cost barriers. Smartphones are ubiquitous, user-friendly and come already equipped with increasingly high-resolution cameras. Building software that capitalizes on existing smartphone hardware allows users to capture reliable, objective, non-invasive wound images and parameters that contribute to better wound assessment and management.

This study measured the length, width and surface area of 45 patient wounds by three different expert raters at the Wound Care Centre at the Women’s College Hospital (Toronto, Canada) using a smartphone application called Swift Wound and developed by Swift Medical. Here, we measure the inter-rater reliability of this tool. In addition, two expert raters using the ruler method measured the wounds of another cohort of 42 patients and an inter-rater reliability for this method was also calculated. We also investigated whether expertise in wound care affected variations in wound measurements. To test this, we recruited three non-expert raters with no wound care training, who then measured the length, width and area of 12 plastic model wounds. The inter-rater reliability of these measurements was evaluated. In addition, accuracy measurements were determined by comparing the Swift Wound app to a digital planimeter of a known accuracy on 15 photos of plastic model wounds. Finally, given the growing body of literature supporting the use of patient home monitoring with infrared thermometers [10–15], as well as a recent study by Fierheller and Sibbald that demonstrated the relationship between increased periwound skin temperature by more than 2° F and local wound infection [16], an infrared FLIR^™^ camera was also integrated into the Swift Wound app and temperature measurements were compared to that of the clinically accepted reference thermometer Exergen DermaTemp 1001, which has been utilized in several peer-reviewed studies [10–15], to determine if an infrared camera attached to a mobile phone can detect localized increases in skin surface temperature comparable to scientific grade instruments. Kanazawa et al. have demonstrated high inter-rater and intra-rater reliabilities when obtaining thermal images in a clinical setting using the FLIR^™^ camera attached to a smartphone [17].

With the Swift Wound app, clinicians can deploy highly reliable and accurate detailed wound photography and thermal imaging in non-subjective assessments at the point-of-care using their smartphone; here we have effectively brought the accuracy and inter-rater reliability of proprietary medical imaging technology to the ubiquitous smartphone.

## Methods

### Swift Wound technology

The Swift mobile app is a medical device that photographs and documents the progression of wound healing over time. The app runs on Apple^®^ iPhone devices with iOS version 8 and above, and includes functions for capture, measurement, documentation, and review of external wounds. All patient data is held secure and encrypted. The Swift Wound app is both HIPPA (Health Insurance Portability and Accountability Act of 1996 U.S.) and PHIPPA (Personal Health Information Protection Act of 2004, Canada) compliant.

For this study, iPhone 6 devices running iOS version 8.4 were used.

Swift also interfaces with off-the-shelf thermal cameras, such as the FLIR One^™^ device, to simultaneously capture far-infrared thermal images of a wound, allowing the practitioner to evaluate average absolute temperatures and temperature differences within and around the wound. The FLIR One^™^ camera captures low-resolution thermal images that can be used to compute aggregate temperature statistics. The FLIR One^™^ device was used in this study for all thermal images and data.

**Fig 1:**
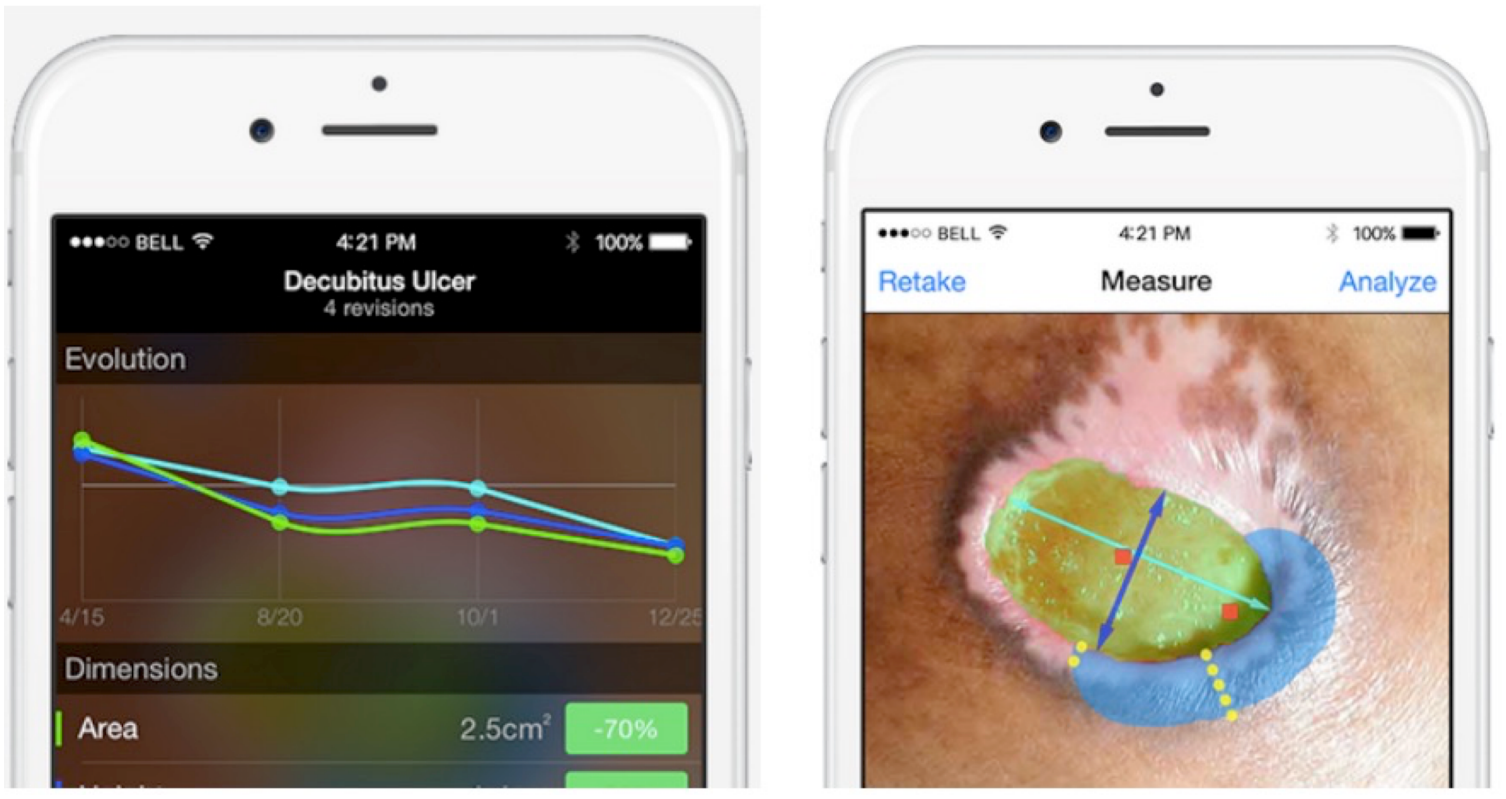
Left: Example of tracking changes in wound size over time using the Swift Wound app. Right: Example of the touch screen interface for measuring the area of a wound using the Swift Wound app. The green region represents the projected surface area of the wound. The dark blue line represents the maximum length, here at a pre-set orientation. The light blue line represents the maximum width perpendicular to length. The blue region represents undermining and the yellow dashed lines represent locations and extent of tunneling. Fig 1 was reprinted from www.swiftmedical.io under a CC BY license, with permission from Swift Medical Inc., original copyright 2015.

To measure and assess a wound, practitioners affix one of Swift’s small 1 cm diameter HealX^™^ adhesive reference marker next to the wound prior to capturing the image. The marker serves as a scale and color reference in images taken of the wound. The practitioner identifies the wound margin on the image, and the true area (cm^2^), and length and width (cm) are computed. The practitioner can opt to compute using the longest length and longest perpendicular width, or a user-selected length orientation (e.g. toward the head) and longest perpendicular width. The practitioner can annotate the wound image with wound depth, and the location and size of any tunneling and undermining (Fig 1).

The Swift Wound app allows practitioners to review a historical timeline of images for a given patient and wound, observing directly the wound’s progression over time, as well as see objectively the orientation and location of the wound margin (and length and width) used in prior images; aiding the acquisition of consistently captured images. This capability was not used as part of this study as the raters were blinded to each other’s measurements, to ensure accurate evaluation of inter-rater reliability.

### Swift Wound app measurements

The Swift Wound app was developed in collaboration with the author (SCW) to optimize its clinical utility. To determine the inter-rater reliability of the smartphone application (Swift Wound app), each rater independently measured the length, width and surface area of 45 wounds on separate smartphone devices using the Swift Wound app. To calibrate wound size and colour, raters placed a HealX^™^ reference marker on the skin next to the wound and took a photo of it with the Swift Wound app. Photos were taken from a distance of 20 cm to 30 cm, and perpendicular to the wound surface to the extent practical, in accordance with instructions provided with the Swift Wound app. Raters then loosely traced the wound directly on the digital photo with their finger. The Swift software identifies the wound edges automatically based on colour models and energy minimization methods, however computer-assisted identification of wound edges may not correlate with how the rater would define the wound’s edge. Therefore, the decision to accept the software’s identification of wound edges or to make manual adjustments to the wound perimeter was left up to the rater as this best reflects how users will interact with the app. The Swift software then, based on the identified wound perimeter, automatically calculates the maximum vertical length and maximum horizontal width, (perpendicular to length), as well as the true area of the wound (Fig 1) by digital planimetry methods.

### Expert measurements

A total of 124 patients attending the Wound Care Centre at Women’s College Hospital (Toronto, Canada) participated in this study. All the patients were receiving treatment for the management of chronic wounds related to diabetes (such as diabetic foot ulcers), venous insufficiency and pressure ulcers. Written informed consent was obtained from each patient participant and the signed consent forms were stored. The Research Ethics Board of Women’s College Hospital approved this consent procedure. This study protocol conformed to the ethical guidelines of the 1975 Declaration of Helsinki as reflected in approval by the Research Ethics Board of Women’s College Hospital.

Three expert raters in clinical wound care were assigned to measure 45 wounds using the smartphone application. These included one physician, one medical student and one nurse. Two additional expert raters, who were nurses, measured an additional 42 wounds using the ruler method. The five raters for this each had between 4–10 years of experience in wound management at the Wound Care Clinic at Women’s College Hospital (Toronto, Canada). Extended visit times and patient discomfort were limited by replicating app and ruler measurements in separate groups of patients. Measurements were made after the wound had been cleansed and debrided and were recorded before the next rater repeated the measurement. All raters were blinded to each other’s assessments. Only patients with a single wound were included in this study to ensure that raters measured the same wound.

### Ruler measurements

To assess the inter-rater reliability of the ruler method, 2 nurses, each with over 10 years of experience in wound care, measured what they perceived to be the longest length (head-to-toe) and longest perpendicular width (side-to-side) of 42 wounds using a disposable 17-cm paper ruler placed directly on the patient’s skin. Each rater was provided a separate table to record the length and width of the wounds. Wounds larger than 17 cm in either dimension were excluded.

### Non-expert measurements

We sought to determine the inter-rater reliability of the Swift Wound app measurements taken by wound-care novices (non-experts), who are familiar with the Swift Wound app. We recruited three non-expert raters who measured the maximum length, width and surface area of 12 different plastic wounds (Pressure Injury Staging Model^™^, VATA Inc., Canby, U.S.A.) using the Swift Wound app, as described above. Inter-rater reliability measurements were then calculated for this data.

### Accuracy of smartphone application surface area measurements

To assess the accuracy of the Swift Wound app, it was used to calculate the area of 15 photos of plastic wounds, with clearly defined margins and surface areas up to 60 cm^2^, taken from wound models (Pressure Inury Staging Model^™^, Wilma Wound Foot^™^ and Seymour II^™^ Wound Care Model, VATA Inc., Canby, U.S.A.), this process was repeated three times. Each wound edge was also traced three times with the tip of a digital planimeter (Placom KP-90N, Koizumi Sokki Mfg.Co.Ltdl, Nagaoka-Shi, Japan) and a numeric display on the planimeter gave a direct read-out of the plane area enclosed by the ulcer tracing. The planimeter has an accuracy of ±0.2% and a resolution of 0.1 cm^2^. We used a 2 (app vs planimeter) x 3 (epoch) mixed ANOVA with epoch serving as the repeated measure to calculate whether measurements were a) stable across episodes within technique, and b) differed by technique. A significant interaction term would indicate that one technique became more or less reliable with repeated measurements than the other.

### Temperature measurements

To assess the performance of the Swift Wound app for measuring surface skin temperatures compared to the clinically accepted reference thermometer Exergen DermaTemp 1001, the difference measured between the wound temperature and the contralateral limb temperature for both the Swift Wound app/FLIR^®^ infrared camera and a standard temperature probe. One rater measured 37 wounds and contralateral limbs using both techniques and the differences in temperature were compared using a paired samples t-test.

### Statistical analysis

Reliability was determined using intraclass correlation coefficients (ICC) calculated with the psych package in R (R Core Team, 2015; Revelle & Revelle, 2015)[18]. Internal reliability was interpreted to be excellent for ICCs = 0.80–1.00. Accuracy measurements were determined by assessing differences in surface area measurements of photos of plastic wounds between planimetry of a known accuracy and the Swift Wound app using a 2 (app, planimeter) x 3 (measurement 1, measurement 2, measurement 3) mixed ANOVA with measure repeated within assessor. Temperature differences between the periwound on one limb and the contralateral limb measured using the FLIR^®^ infrared camera/Swift Wound app and the clinically accepted reference thermometer were compared using a paired samples t-test to determine whether a difference existed between the two methods. All data available as supporting information (S1 Raw data).

## Results

### Inter-rater reliabilities of the app exceed ruler measurements

**Fig 2:**
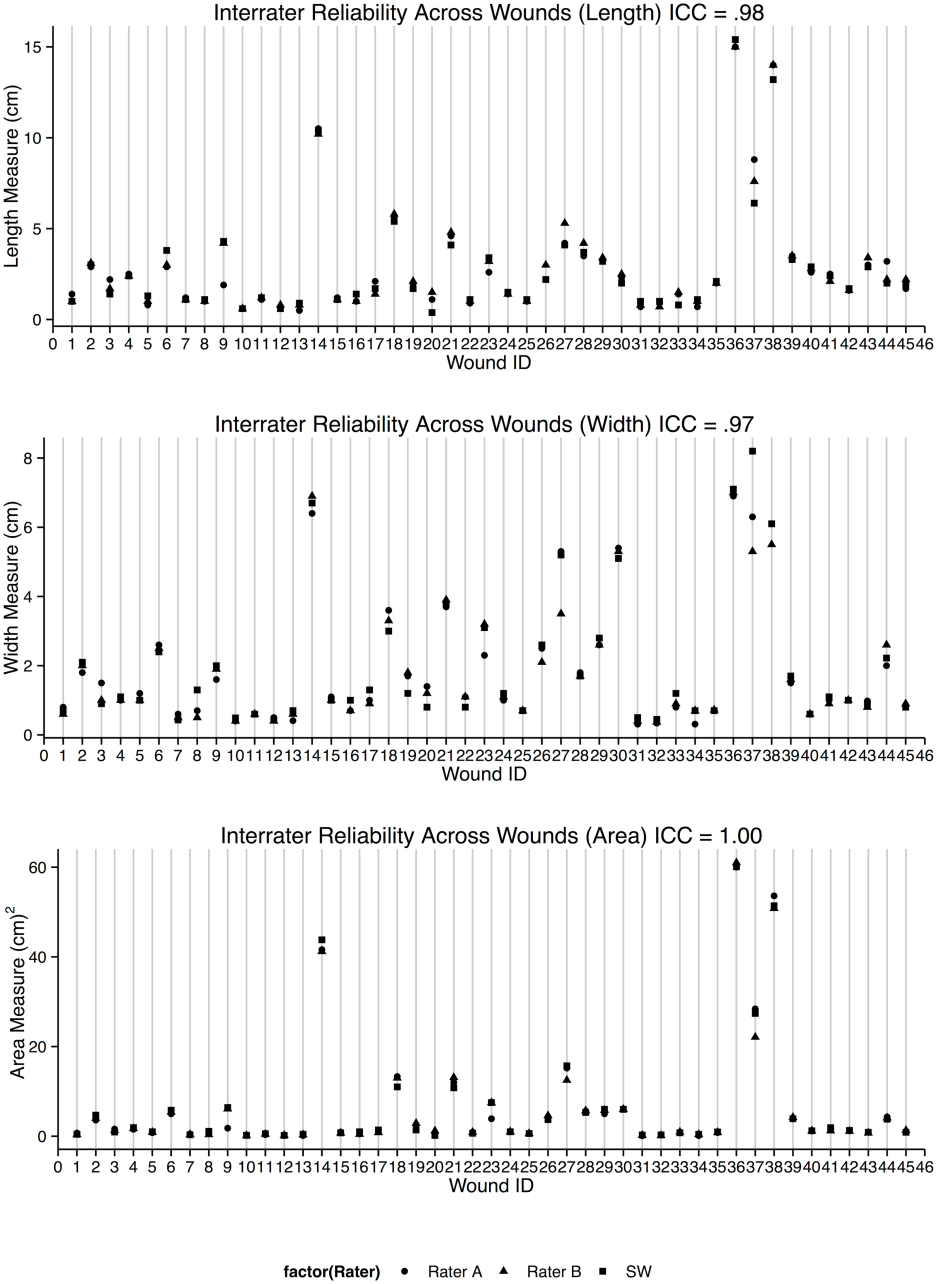
Inter-rater reliabilities between length, width and surface area measurements using the Swift Wound app (ICCs = 0.98. 0.97 and 1.00 respectively).

**Fig 3:**
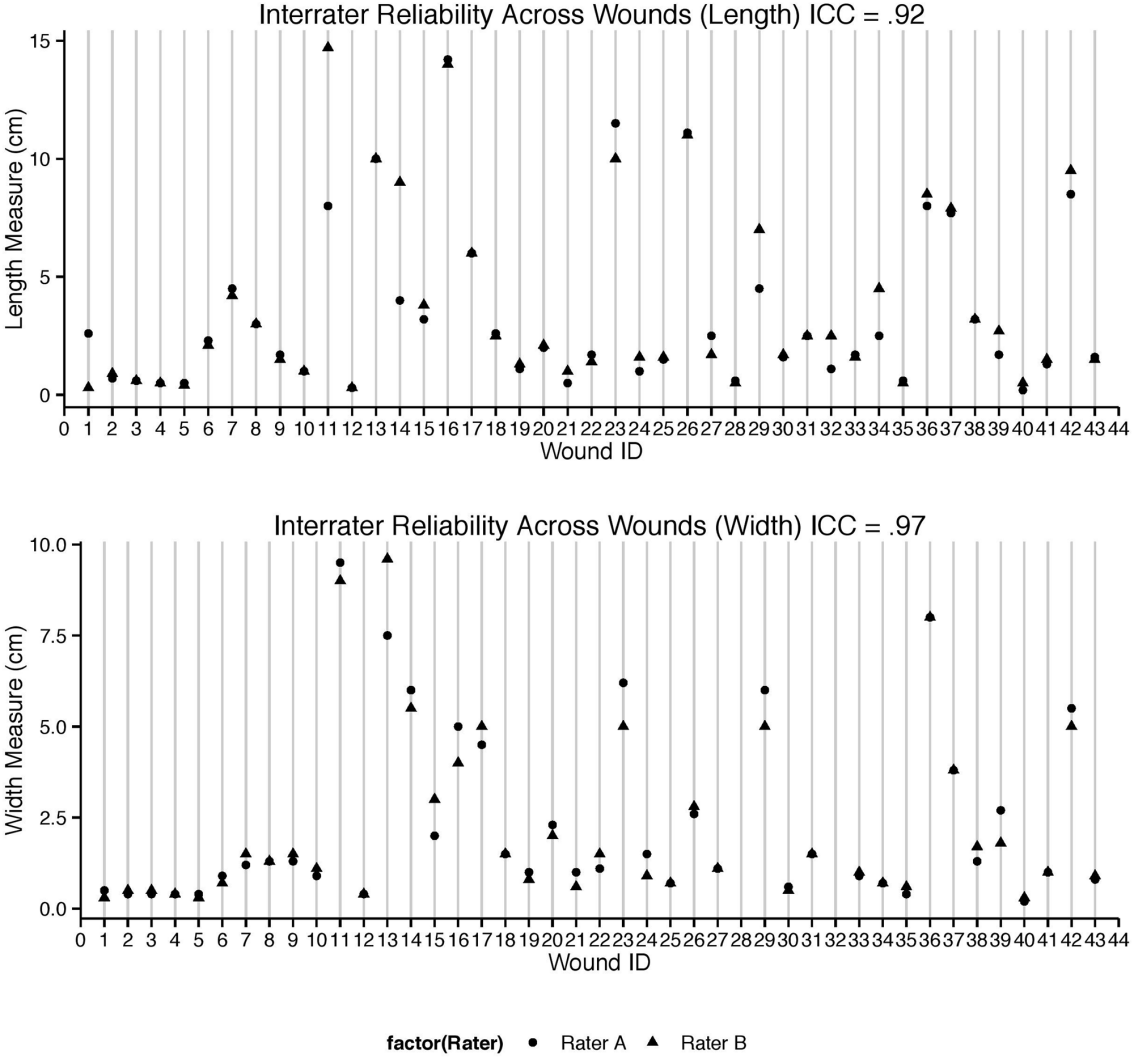
Inter-rater reliabilities between length and width using the ruler method (ICCs– 0.92 and 0.97 respectively). Note that eliminating case 11 changes the ICC for length from 0.92 to 0.95, CI (0.92-.097), eliminating this case did not change the ICC estimate for width: 0.97, CI (0.94–0.98).

High inter-rater reliabilities were observed using the Swift Wound app across all wound sizes ranging from 0.2 cm^2^ to 60 cm^2^ (ICCs = 0.98, 0.97 and 1.00 for length, width and surface area measurements respectively, Table 1 and Fig 2). The greatest agreement was observed in calculating the surface area of wounds (ICC = 1.00, 95% CI = 0.99–1.00, Table 1 and Fig 2). Lower reliability coefficients were observed when using the ruler method to measure perceived maximum length and width (ICCs = 0.92, 95% CI = 0.86–0.96 and 0.97, 95% CI = 0.95–0.99 for length and width measurements respectively, Table 1 and Fig 3) in 42 wounds with lengths and widths ranging from 0.2–14.7 cm and 0.2–9.6 cm respectively.

**Table 1:** Inter-rater reliability of measurements of wounds using the Swift Wound app and the ruler.

ICC			
Tool	Length	Width	Surface Area
**App (n = 45)**	0.98, 95% CI (0.98–0.99)	0.97, 95% CI (0.95–0.98)	1.00 95% CI (0.99–1.00)
**Ruler (n = 42)**	0.92, 95% CI (0.86–0.96)	0.97, 95% CI (0.95–0.99)	
**App by Novice (n = 12)**	0.99, 95% CI (0.99–1.00)	0.99, 95% CI (0.97–1.00)	0.99, 95% CI (0.99–1.00)

#### Non-expert inter-rater reliabilities of wound measurements using the Swift Wound app are high

**Fig 4:**
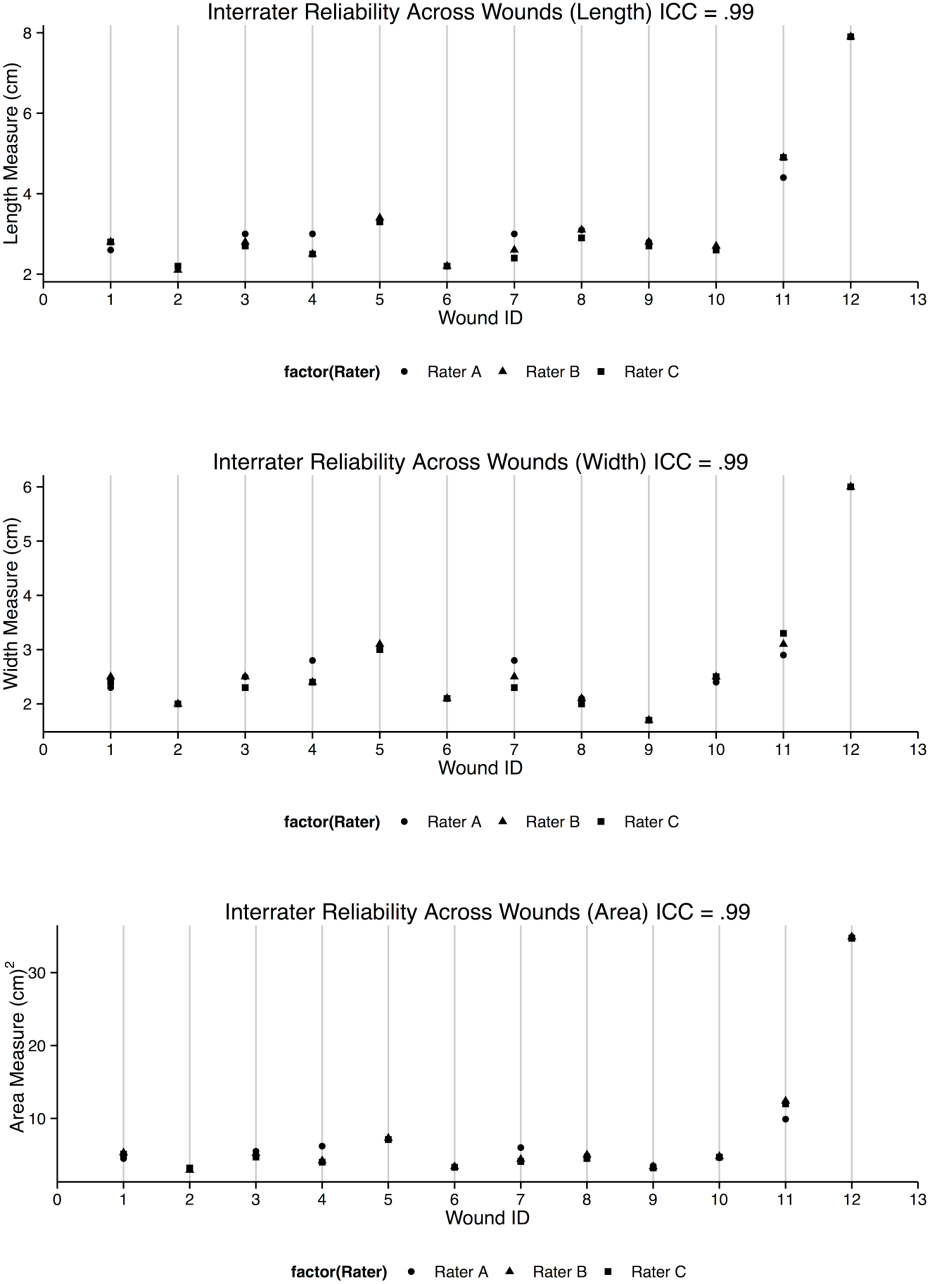
Interrater reliabilities for length, width and surface area using the Swift Wound app by non-experts (ICC = 0.99, 0.99 and 0.99 respectively).

High inter-rater reliabilities were observed across all measurements of length, width and surface area (ICCs = 0.99, 95% CI = 0.99–1.00, Table 1 and Fig 4), similar to the high reliability coefficients observed in app measurements of patient wounds by clinical wound experts.

#### Swift Wound app area measurements are accurate

**Fig 5:**
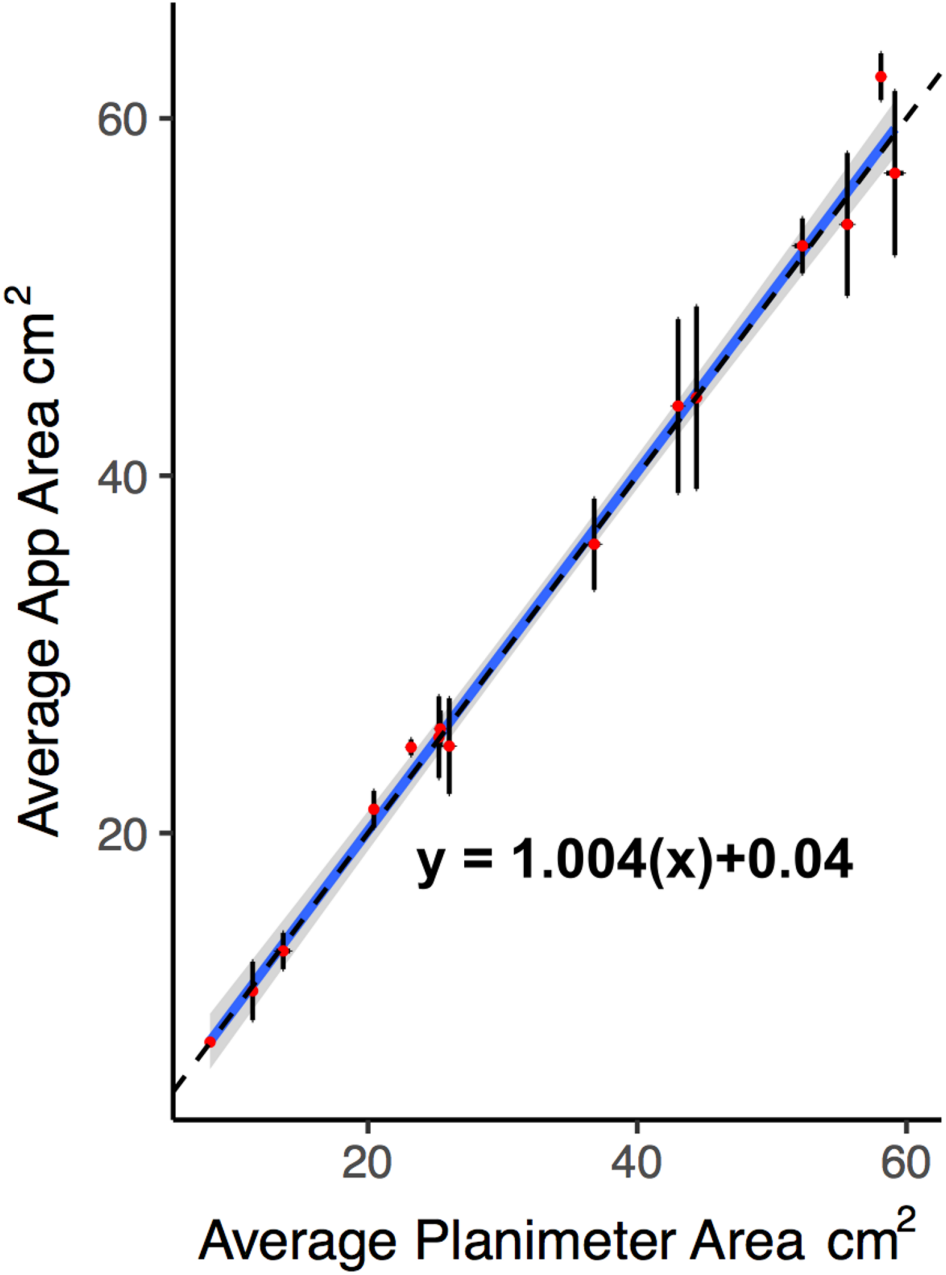
Accuracy of the Swift Wound app. Planimeter area measurements predicting Swift Wound app area measurements for 15 photos of plastic wound shapes. The dashed line represents the line of unity (i.e. a perfect fit). Points represent the average across three measures ± 95% CI.

Given the high repeatability between non-expert wound measurements using the Swift Wound app, we then assessed the accuracy of the app by using it to calculate the areas of 15 photos of plastic wound shapes with areas up to 60 cm^2^, three times and compared these measurements to three area measurements taken using a digital planimeter of known accuracy. The reliability measures per wound shape for the app and the planimeter were very high (ICC = 0.998 CI [0.996–0.999], S1 Fig). A comparison of the digital planimeter area measurements to the Swift Wound app measurements show that none of the main effects nor interactions reached significance, all *p* values > 0.13 (S1 Table, S2 Fig), suggesting that the Swift Wound app performs comparably to the digital planimeter (Fig 5).

#### Swift Wound and FLIR^®^ infrared camera temperature measurements are comparable to scientific grade instruments

**Fig 6:**
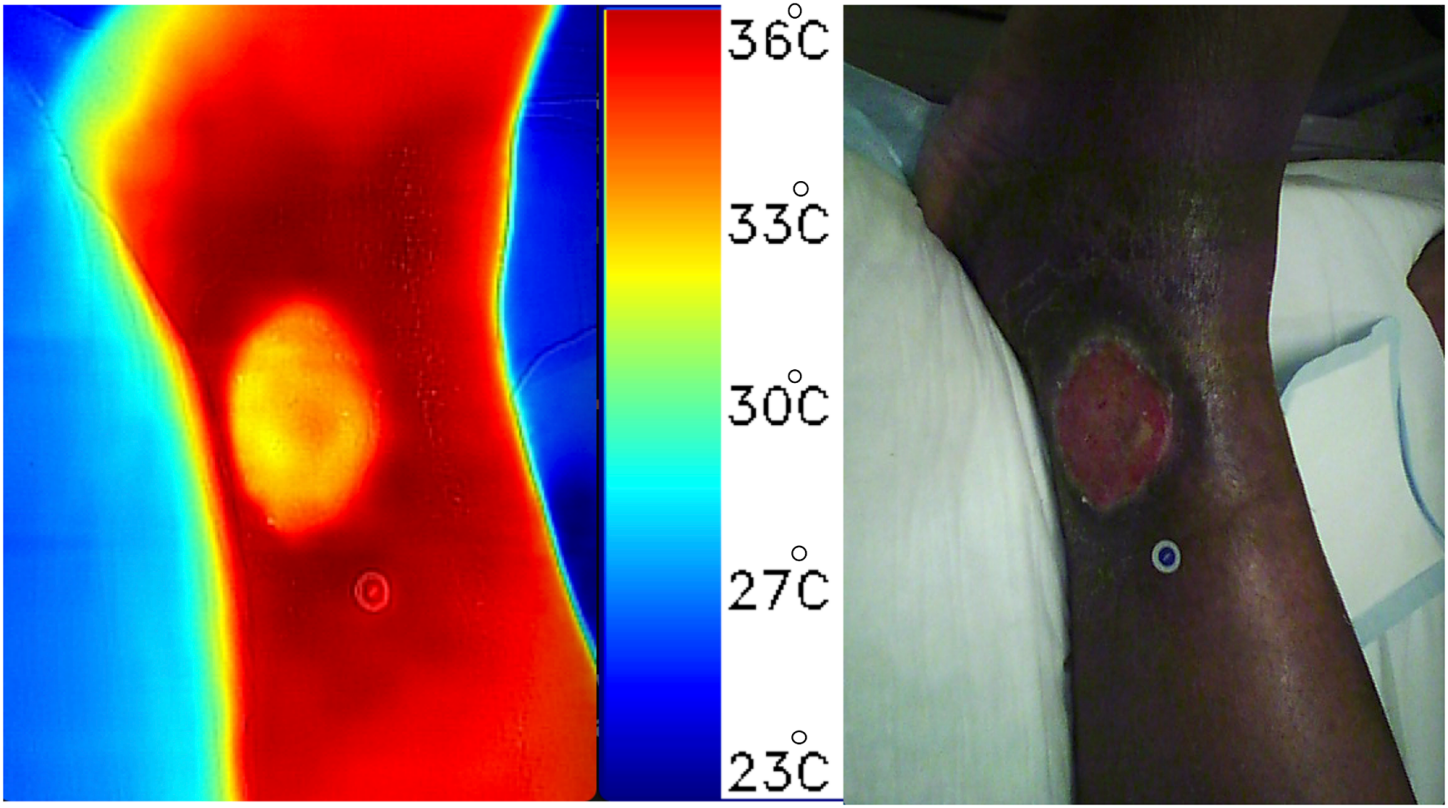
Example of an infrared image of an ulcer on the medial malleolus, taken using the Swift Wound app and a FLIR^™^ infrared camera. Left: Infrared image displaying heat distribution around a venous ulcer on the medial malleolus with a temperature scale. Right: Corresponding white light photograph of the ulcer.

To assess the performance of the Swift Wound app for measuring skin surface temperature, we computed the difference measured between the wound bed and the contralateral limb skin temperature for both the clinically accepted reference thermometer Exergen DermaTemp 1001 and the Swift Wound app using a FLIR^®^ infrared camera (Fig 6). The difference between the two methods was non-significant using a paired t-test, *t*(34) = 1.29, p = 0.21 (S2 Table).

## Discussion

In this study, we demonstrate that the Swift Wound app provides reliable (ICC = 0.97–1.00) and accurate measurements of wounds. The standard ruler method also yields fairly reliable length and width measurements, (ICC = 0.92–0.97), but is less robust than the app. When using the ruler method, typically only simple length and width measurements are recorded in the clinic because multiplying the two measurements to obtain a rough estimate of a rectangular area has been shown to introduce large variability and inaccuracies [5, 6]. Length and width measurements alone, however, do not capture islands of healing within the wound bed or healing at the wound edge that are independent of maximum length and width dimensions. Furthermore, ruler-based measurements have been shown to become less reliable in larger, irregular wounds [5, 6]. Here we demonstrate that the inter-rater reliability of the Swift Wound app is unaffected by wound size and that the Swift Wound app is accurate for the different wound shapes tested in our study. Follow-up studies may wish to collect a larger sample of wound shapes and sizes to extend the results we present here. Ultimately, changes in wound area, as calculated by the Swift Wound app, provide a better assessment of wound healing over time [19] compared to simple ruler length and width measurements. Furthermore, unlike the ruler method, the app is a non-contact tool that measures wound dimensions quickly and easily without touching the wound bed.

There was also no statistical difference between the differences in temperature measurements for the FLIR^®^ infrared camera integrated with the Swift Wound app when compared to that of the clinically accepted reference thermometer, demonstrating that the infrared FLIR^®^ camera can detect localized changes in skin surface temperature comparable to scientific grade instruments; this additional feature enables wound care practitioners to detect signs of inflammation or infection [17, 20] in addition to tracking changes in wound size over time.

Of note, high inter-rater reliability was observed among clinical experts as well as non-experts, suggesting that patients and their caregivers can also use this tool for home monitoring. The authors previously demonstrated that being able to visualize difficult-to-see wounds using digital photography can improve patient satisfaction, adherence to treatment and involvement in their own wound care [21].

A limitation of this study is that the plastic model wounds used by the non-expert raters had clear margins. By contrast, in real patient wounds, (which were measured by the expert raters), the wound edge is often not well defined. Previous studies have demonstrated that variation between wound measurements is largely due to differences in subjective identification of the wound edge[6, 22]. This is a challenge for traditional as well new digital wound imaging tools and is addressed in two ways by the Swift Wound app. Firstly, the Swift Wound app automatically identifies wound edges with clear colour contrast, providing an objective demarcation of the wound margins to the rater, who may accept, reject, or modify the identified margins. However, when there is surrounding inflammation and poor colour contrast, automatic identification of the wound edges may not be possible for the software, making the ability to manually identify wound margins necessary. A second feature of the Swift Wound app allows clinicians to retrieve and overlay previous traces of the wound edge on newly acquired images. This eliminates the need for different raters to redefine the wound edge at each appointment and thus reduce inconsistencies across visits.

Several wound measurement devices have been developed recently to address the challenges of wound imaging and measurement [8, 9, 23]. Costs however continue to be an important factor, limiting dissemination of these tools in clinics and in community care teams. A solution we propose is to take advantage of recent advances in mobile technology by developing an app that is an inexpensive, easy-to-use, reliable and accurate tool for wound measurement. This app is further able to photo-document and track wound healing over time. Finally, we suggest that there are several additional benefits to patients, clinicians, researchers and policy makers inherent in adopting this platform. This tool has the potential to greatly increase the efficiency of assessing and documenting changes in wound healing and exchanging this information between stakeholders. To our knowledge, this is the first study to validate clinically a mobile app for wound size and temperature measurement. Importantly, the Swift Wound app can be used reliably by both expert and non-experts in wound care. In principle, a mobile wound care application can be used in a wide range of situations, whether a primary care environment, a telemedicine consultation, or home monitoring. This technology has the potential to significantly impact clinical management and patient outcomes.

## Supporting information

S1 Fig Reliability measures per wound for the app (circle) and planimeter (square). ICC values for the planimeter were 1 with no detectable variation. ICC values for the App were 0.998 CI [0.996–0.999].
(PDF)

S2 Fig Descriptive plot. (PDF)

S1 Table Repeated measures ANOVA within subjects effects (A) and between subjects effects (B). (PDF)

S2 Table Temperature data. The difference between the two methods was non-significant using a paired t-test, *t*(34) = 1.29, p = 0.21.
(PDF)

S1 Raw Data (XLSX)
