# Supplementary material for: Point-of-care wound visioning technology: Reproducibility and accuracy of a wound measurement app
Source: PLoS One. 2017 Aug 17;12(8):e0183139. doi: 10.1371/journal.pone.0183139 (PMC5560698; doi:10.1371/journal.pone.0183139)
Supplement: S1 Fig — ICC values for the planimeter were 1 with no detectable variation. ICC values for the App were 0.998 CI [0.996–0.999]. (PDF) [file pone.0183139.s001.pdf]

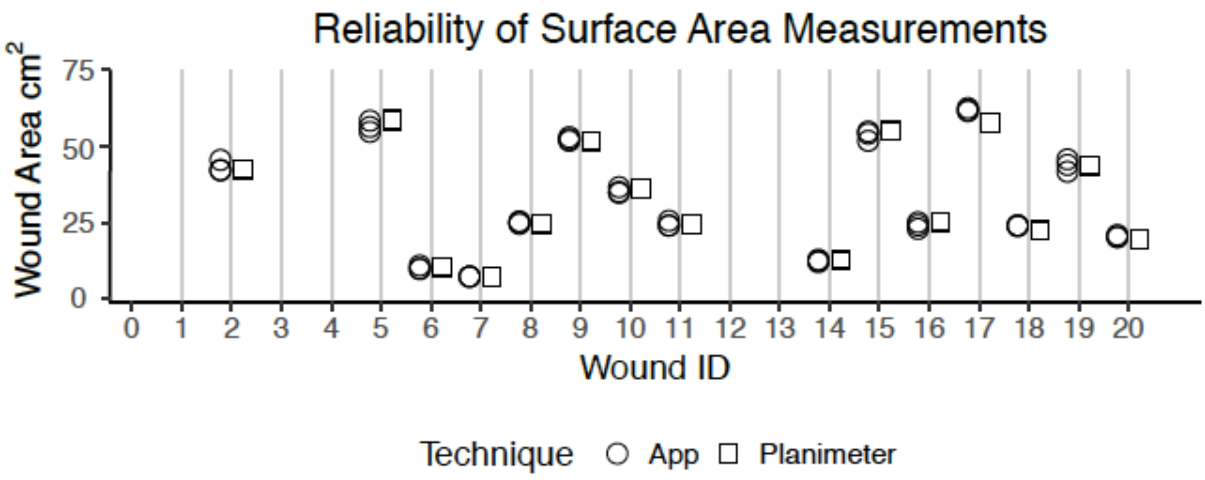

S1 Fig. **Reliability measures per wound for the App (circle) and planimeter (square).** ICC values for the planimeter were 1 with no detectable variation. ICC values for the App were 0.998 CI [0.996 – 0.999].
