# Supplementary figures and images for: Point-of-care wound visioning technology: Reproducibility and accuracy of a wound measurement app
Source: PLoS One. 2017 Aug 17;12(8):e0183139. doi: 10.1371/journal.pone.0183139 (PMC5560698; doi:10.1371/journal.pone.0183139)

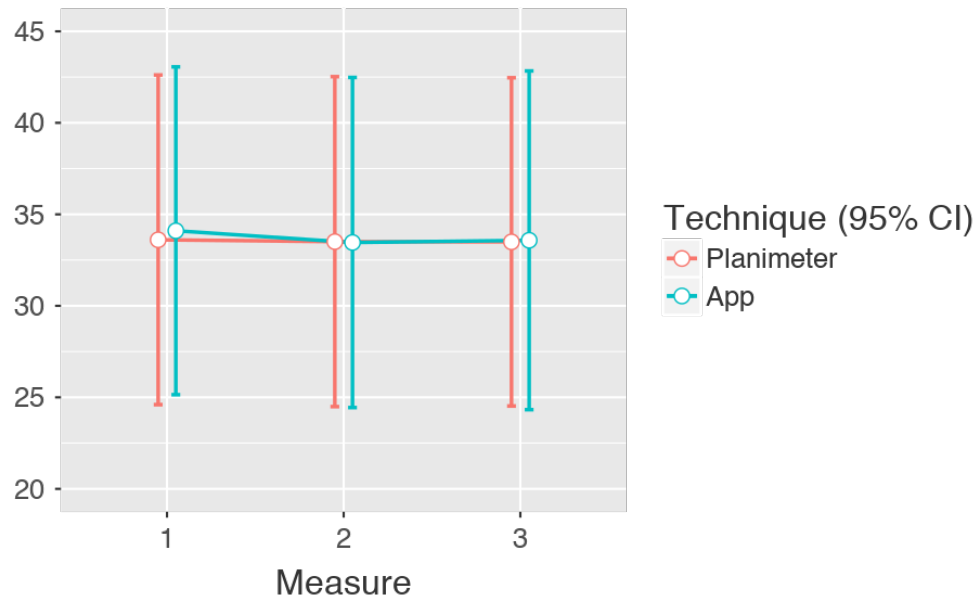

**S2 Fig.** Descriptive Plot

Supplement: S2 Fig — (PDF) [file pone.0183139.s002.pdf]
