# Supplementary material for: Point-of-care wound visioning technology: Reproducibility and accuracy of a wound measurement app
Source: PLoS One. 2017 Aug 17;12(8):e0183139. doi: 10.1371/journal.pone.0183139 (PMC5560698; doi:10.1371/journal.pone.0183139)
Supplement: S1 Table — (PDF) [file pone.0183139.s003.pdf]

A. Within Subjects Effects

	Sum of Squares	df	Mean Square	F	p	partial η^2
Technique	0.711	1	0.711	0.212	0.652	0.015
Residual	46.909	14	3.351			
Measure	2.400	2	1.200	2.154	0.135	0.133
Residual	15.603	28	0.557			
Technique ✻ Measure	1.187	2	0.593	0.970	0.391	0.065
Residual	17.123	28	0.612			

Note. Type 3 Sums of Squares

B. Between Subjects Effects

	Sum of Squares	df	Mean Square	F	p	partial η^2
Residual	25987	14	1856			

Note. Type 3 Sums of Squares

S1 Table. Repeated measures ANOVA within subjects effects (A) and between subjects effects (B).
