# Supplementary material for: Point-of-care wound visioning technology: Reproducibility and accuracy of a wound measurement app
Source: PLoS One. 2017 Aug 17;12(8):e0183139. doi: 10.1371/journal.pone.0183139 (PMC5560698; doi:10.1371/journal.pone.0183139)
Supplement: S2 Table — The difference between the two methods was non-significant using a paired t-test, t(34) = 1.29, p = 0.21. (PDF) [file pone.0183139.s004.pdf]

Thermal Data Temperature (Means & SEs)

	Probe Temp F	Flir Temp F	Probe Contra Temp F	Flir Contra Temp F
Mean	90.24	91.65	91.05	93.40
SD	5.48	6.27	5.65	5.58
SE	0.90	1.03	0.94	0.94

S2 Table. Temperature Data. The difference between the two methods was non-significant using a paired t-test, $t(34) = 1.29$, $p = 0.21$.
